# Association between occupational stress, social support at work, and physical activity in outsourced workers

**DOI:** 10.47626/1679-4435-2022-804

**Published:** 2023-02-13

**Authors:** Roberta Arruda Alves, Thaísa Alves Penna, Vitor Barreto Paravidino, Aldair J. Oliveira

**Affiliations:** 1 Universidade Salgado de Oliveira, Niterói, RJ, Brazil; 2 Universidade do Estado do Rio de Janeiro, Rio de Janeiro, RJ, Brazil; 3 Universidade Federal Rural do Rio de Janeiro, Seropédica, RJ, Brazil

**Keywords:** occupational stress, mental health, physical exercise, interpersonal relationships, estresse ocupacional, saúde mental, exercício físico, relações interpessoais

## Abstract

**Introduction:**

Psychosocial factors at work can affect individuals’ physical and mental health. In this sense, evidence shows that physical activity and social support at work promote benefits to workers’ health, especially regarding stress reduction.

**Objectives:**

To evaluate the association between occupational stress, social support at work, and weekly frequency of physical activity among outsourced workers.

**Methods:**

This is a cross-sectional study with a convenience sample comprising 182 outsourced workers of both sexes and different positions, aged between 21 and 72 years (39 ± 11.4); the participants answered the Demand-Control-Support Questionnaire for assessing occupational stress and social support at work and the International Physical Activity Questionnaire - Short Form for assessing the frequency of physical activity. The association between constructs was investigated through a Poisson regression. The significance level was set to 5%.

**Results:**

A significant inverse association (p < 0.05) was found between passive work and frequency of physical activity (walking) among women (relative risk 0.70; 95% confidence interval 0.5-0.9), whereas for men, this association was found with the frequency of vigorous-intensity physical activity (relative risk 0.70; 95% confidence interval 0.4-0.9). However, a significant inverse association (p < 0.05) between social support and physical activity was found only among women (relative risk 0.66; 95% confidence interval 0.4-0.9) and for moderate- and vigorous-intensity physical activity (relative risk 0.65; 95% confidence interval 0.4-0.9).

**Conclusions:**

Occupational stress and social support at work are associated with weekly frequency of physical activity. Nevertheless, disparities can be seen between men and women and depending on the intensity of physical activity.

## Abstract (pt)

**Introdução:**

Fatores psicossociais do trabalho podem afetar a saúde física e mental dos indivíduos. Neste sentido, evidências mostram que a atividade física e o apoio social no trabalho promovem benefícios à saúde de trabalhadores, sobretudo na redução do estresse.

**Objetivos:**

Avaliar a associação entre estresse ocupacional, apoio social no trabalho e frequência semanal de atividade física em trabalhadores terceirizados.

**Métodos:**

Estudo transversal com amostra de conveniência de 182 trabalhadores terceirizados de ambos os sexos e de diferentes cargos, com idade entre 21 e 72 anos (39±11,4), os quais responderam ao questionário Demand Control-Support Questionnaire, para avaliar o estresse ocupacional e o apoio social no trabalho, e ao Questionário Internacional de Atividade Física curto, para avaliar a frequência da atividade física. A associação entre os constructos foi investigada através da regressão de Poisson. O nível de significância adotado foi de 5%.

**Resultados:**

Uma associação inversa significativa (p < 0,05) foi encontrada entre trabalho passivo e frequência de atividade física de caminhada para mulheres (risco relativo de 0,70; intervalo de confiança de 95% de 0,5-0,9), enquanto, para homens, foi encontrada com frequência de atividade física vigorosa (risco relativo de 0,70; intervalo de confiança de 95% de 0,4-0,9). Todavia, uma associação inversa significativa (p < 0,05) entre apoio social e atividade física foi encontrada apenas em mulheres (risco relativo de 0,66; intervalo de confiança de 95% de 0,4-0,9) e para intensidade moderada e vigorosa (risco relativo de 0,65; intervalo de confiança de 95% de 0,4-0,9).

**Conclusões:**

Estresse ocupacional e apoio social no trabalho estão associados à frequência semanal de atividade física. No entanto, disparidades podem ser observadas entre homens e mulheres e intensidade de atividade física.

## INTRODUCTION

In recent decades, the workplace has been widely discussed in scientific literature.^1^ The highly competitive environment generated at work affects the health status of individuals, contributing to stress and consequently leading to diseases such as anxiety, depression, and cardiovascular diseases.^2^ Occupational stress originates in the workplace and encompasses matters such as organizational management, working conditions, and quality of interpersonal relationships at work.^3^

Studies on job demands, job control/decision latitude, and social support started being conducted in the 1980s, and an increase in studies investigating occupational stress and its possible influencers has been seen through the years.^4^ Epidemiological studies present different perspectives on the impact of workload on workers’ health.^5^ A fraction of these individuals may present muscular disorders and inadequate posture,^6^ in addition to mental disorders related to occupational demands.^2^ Outsourced services emerged as a response to the need of public and private companies to have efficient and fast production and reduce costs, becoming common practice in Brazil and worldwide. Outsourced workers are usually undervalued and physically overworked, and this can affect their health and quality of life.^7^ However, few studies in the occupational domain focus on this group.

When searching for alternatives that improve employees’ quality of life, physical activity performed during leisure time, household chores, commuting, and at the workplace has been indicated as a primary factor of health promotion.^8^ Studies show that the physical environment, feeling of security, and recreational facilities as well as clubs and courts were positively associated with physical activity, with individuals presenting higher chances of being physically active.^9^ Social support is widely studied and has been shown as one of the determinant factors of physical activity adherence. However, studies that approach the relationship between social support at the workplace and physical activity are still scarce.^10^

Evidence indicates that men are usually more engaged in leisure-time physical activities when compared to women.^11^ Moreover, women are more prone to facing stressor events at work when compared to men.^12^ Current findings show that occupational stress is directly related to mental and physical health and that individuals with high levels of occupational stress present low levels of physical activity.^13^ The most recent World Health Organization (WHO) guidelines advocate that adult individuals (aged between 18 and 64 years), including those with some kind of chronic disease or disability, should do 150 to 300 minutes of moderate-intensity aerobic physical activity or 75 to 150 minutes of vigorous-intensity aerobic physical activity per week, with possible combinations of these intensities throughout the week.^14^

Although investigations on occupational stress, social support at work, and physical activity are evident in the literature,^12,15^ studies that emphasize the frequency of physical activity are still scarce, considering that most of them focus on analyzing the level of physical activity. Therefore, it is important to investigate the weekly frequency of physical activity among the working population, since lack of free time is mentioned as one of the main barriers to physical activity. In addition, few studies focus on investigating possible associations between the aforementioned constructs. Therefore, the aim of this study was to evaluate the association between occupational stress, social support at work, and weekly frequency of physical activity among outsourced workers of Universidade Federal Rural do Rio de Janeiro (UFRRJ).

## METHODS

### STUDY DESIGN

This is a cross-sectional population-based study conducted at the Seropédica campus of UFRRJ. The study was approved by the Research Ethics Committee of Universidade Salgado de Oliveira (Certificate of Presentation for Ethical Appreciation 56224716 2 0000 5289).

### PARTICIPANTS

The study population comprised a convenience sample of 182 outsourced workers of both sexes, most of whom were general services assistants, cleaning assistants, or janitors. The criteria for including participants were being 18 years old or older and an outsourced employee at the university; exclusion criteria were being lent to another institution or on medical leave.

### CONTEXT

Data were collected through a self-administered questionnaire comprising instruments that assessed physical activity, occupational stress, social support at work, and sociodemographic information. The team responsible for data collection included previously trained researchers, and data collection began upon authorization of the supervisors at each sector. Participation in this study was voluntary and all workers were informed of the research procedures. Those who agreed to participate were instructed to read and sign the free and informed consent form, and data were then individually collected in a private location and without interfering with the normal work activities of each sector. For those with a low education level or difficulty understanding the instrument, data collection was performed through an interview, with no interference by the researcher. Data were then stored in an electronic spreadsheet through double data entry in order to minimize possible errors. At first, the data collection procedure took place between June and August 2017; a second phase took place in June and July 2018.

### VARIABLES

In this study, we used occupational stress and social support at work as exposure variables. On the other hand, the frequency of leisure-time physical activity was used as an outcome variable. Moreover, education level and age were used as confounding variables.

### DATA SOURCES AND MEASUREMENTS

Physical activity was investigated through the International Physical Activity Questionnaire (IPAQ) - Short Form. This instrument, validated and reproduced in Brazil by a pilot test by Matsudo et al.,^16^ presents eight items that aim to identify the duration (minutes per session) and frequency (per week) of walking and moderate- and vigorous-intensity physical activities. This study, on the other hand, focused only on the weekly frequency of different types of physical activity. Therefore, the frequency of leisure-time physical activity was classified into four categories: 0 days per week (inactive), 1 to 3 days, 4 to 5 days, and 6 to 7 days per week. As to the type of physical activity, the classification categories were walking, moderate-intensity physical activity, and vigorous-intensity physical activity. Inactive individuals were used as reference in the analyses.

The second instrument used in this study was the Demand-Control-Support Questionnaire (DCSQ), developed in 1988 by Töres Theorell and translated and validated into Portuguese in 2004.^17^ The instrument went through an evaluation by judges and, later, a pilot test was performed for verifying its efficacy and applicability. It is a Likert-type scale used for verifying the level of occupational stress and social support at work among participants. This scale has 17 items: five for assessing the “psychological demands” dimension, six for assessing decision latitude, and six for assessing social support at work. The scale has four answer options: (1) often; (2) sometimes; (3) rarely; and (4) never/almost never. The occupational stress scores were obtained from the sum of the answers to the first two dimensions. Considering psychological demands, the scores varied from 5 to 20, with a cutoff of 14. Individuals who scored between 5 and 14 are classified as having low job demands and those who scored > 14, as with high demands. Regarding decision latitude, the scores varied from 6 to 24, with a cutoff of 17. Workers scoring between 6 and 17 were classified as with low decision latitude and those who scored > 17, with high decision latitude.^18^

Individuals were classified into four categories: low strain (low job demands and high decision latitude), passive job (low job demands and low decision latitude), active job (high job demands and high decision latitude), and high strain (high job demands and low decision latitude). The “low strain” category was used as reference in the analyses. As to social support at work, the cutoff was 17, where those scoring ≤ 17 were classified as having low social support whereas those scoring > 17 were classified as having high social support. The “low support” category was used as reference in the analyses.

The population’s age was categorized into four groups: 20 to 35 years old, 36 to 59 years old, and 60 years old or older. Education levels were grouped into four categories: lower secondary education (incomplete or complete), upper secondary education (incomplete or complete), and higher education (incomplete, complete, or graduate education).

### STATISTICAL METHODS

Descriptive analyses were performed including frequencies and percentages for categorical variables and means and standard deviations for continuous variables. As to our inferential approach, we performed a Poisson regression for assessing the weekly frequency of physical activity according to occupational stress and social support at work. Relative risk (RR) and the respective 95% confidence intervals (95%CI) were estimated for the crude and adjusted models. The significance level was set to 5%. The age and education level variables were included in the adjusted model. All analyses were stratified by sex and performed using R software, version 3.5.6.

## RESULTS

Out of 182 outsourced technical/administrative workers, more than half were aged between 36 and 59 years. Approximately 50% of women and 52.8% of men had incomplete or complete upper secondary education. Considering occupational stress, 47.6% of the women were classified as passive workers, that is, had low job demands and low decision latitude; similarly, 46.1% of the men were included in the same occupational stress category. While 91.3% of the men were classified as having high social support at work, 85.4% of the women reached high levels in this resource. When it comes to physical activity, 40.9% of the women walked four to five times a week; conversely, 28.6% of the men had the same frequency of walks. Only 7.9% of the women did vigorous-intensity physical activity six to seven times a week, while 19.1% of the men had the same frequency of vigorous-intensity physical activity. More details can be seen in Table 1.

**Table 1:** Descriptive characteristics of the sample according to sex

Variables	Number of observations n (%)					
	Women (n = 89)			Men (n = 93)		
Age (years)						
20-35	39 (43.8)			31 (33.3)		
36-59	47 (52.8)			55 (59.1)		
≥ 60	3 (3.4)			7 (7.5)		
Education level						
Lower secondary education	30 (33.7)			22 (24.2)		
Upper secondary education	43 (48.3)			48 (52.8)		
Higher education	16 (18.0)			21 (23.1)		
Job strain						
Low strain	19 (22.6)			21 (23.6)		
Passive work	40 (47.6)			41 (46.1)		
Active work/high strain	25 (29.8)			27 (30.3)		
Social support at work						
Low support	13 (14.6)			8 (8.7)		
High support	76 (85.4)			84 (91.3)		
Weekly frequency	PA, walking	Moderate-intensity PA	Vigorous-intensity PA	PA, walking	Moderate-intensity PA	Vigorous-intensity PA
0	16 (18.2)	14 (15.7)	35 (39.3)	18 (19.8)	17 (18.5)	24 (27.0)
1-3	13 (14.8)	36 (40.5)	22 (24.8)	18 (19.8)	32 (34.8)	31 (34.8)
4-5	36 (40.9)	16 (18.0)	25 (28.1)	26 (28.6)	25 (27.1)	17 (19.1)
6-7	23 (26.1)	23 (25.8)	7 (7.9)	29 (31.9)	18 (19.6)	17 (19.1)

Higher education = incomplete or complete higher education or graduate education; lower secondary education = incomplete or complete lower secondary education; PA = physical activity; upper secondary education = incomplete or complete upper secondary education.

Considering occupational stress, we identified a significant inverse association (p < 0.05) in women classified as passive workers with the weekly frequency of physical activity (walking) when compared with those with low job strain, which were considered the best scenario (RR = 0.70; 95%CI 0.5-0.9). Similarly, an inverse association was observed in men who were passive workers and did vigorous-intensity (RR = 0.70; 95%CI 0.4-0.9) and moderate-intensity physical activity (RR = 0.71; 95%CI 0.5-0.9) when compared with men with low job strain.

In women, a positive association was seen between active work and high job strain and the frequency of vigorous-intensity physical activity (RR = 1.51; 95%CI 1.0-2.2). Conversely, men in the same occupational stress category displayed a significant inverse association (p < 0.05) with the frequency of vigorous-intensity physical activity (RR = 0.76; 95%CI 0.5-1.0). We also observed a significant inverse association in men (p < 0.05) between active individuals with high job strain and walking (RR = 0.67; 95%CI 0.5-0.9).

As to social support at work and frequency of physical activity, a significant association was found only among women. We observed a significant inverse association (p < 0.05) among those classified as having high social support at work and the frequency of moderate-intensity physical activity compared with those who had low support (RR = 0.66; 95%CI 0.4-0.9). Similarly, we found a significant inverse association (p < 0.05) among women with high social support at work and the frequency of vigorous-intensity physical activity compared with those classified as having low social support (RR = 0.65; 95%CI 0.4-0.9). More details are presented in Tables 2 and 3.

**Table 2:** Relative risk and their respective 95% confidence intervals for associations between occupational stress/social support at work and frequency of physical activity (PA) among women

Job strain	Weekly frequency of PA					
	Women RR (95%CI)					
	PA, walking		Moderate-intensity PA		Vigorous-intensity PA	
	Crude model	Adjusted model	Crude model	Adjusted model	Crude model	Adjusted model
Karasek’s dimensions						
Low strain	1	1	1	1	1	1
Passive work	1.11 (0.8-1.4)	1.07 (0.8-1.4)	1.05 (0,7-1,4)	1.03 (0.7-1.3)	1.07 (0.7-1.5)	1.10 (0,7-1,6)
Active work/high strain	1.17 (0.8-1.5)	1.13 (0.8-1.5)	0.97 (0,7-1,3)	0.92 (0.6-1.3)	1.40 (0,9-2,0)	1.51 (1,0-2,2)
Social support at work						
Low	1	1	1	1	1	1
High	0.88 (0.6-1.1)	0.76 (0.5-1.0)	0.77 (0.5-1.0)	0.66 (0.4-0.9)	0.64 (0.4-0.9)	0.65 (0.4-0.9)

RR and respective 95%CI for the Poisson regression models; adjusted model: age and education level. All statistically significant associations are highlighted in bold (p < 0.05).

95%CI = 95% confidence interval; PA = physical activity; RR= relative risk.

**Table 3:** Relative risk and their respective 95% confidence intervals for associations between occupational stress/social support at work and frequency of physical activity among men

Job strain	Weekly frequency of PA					
	Men RR (95%CI)					
	PA, walking		Moderate-intensity PA		Vigorous-intensity PA	
	Crude model	Adjusted model	Crude model	Adjusted model	Crude model	Adjusted model
Karasek’s dimensions						
Low strain	1	1	1	1	1	1
Passive work	0.75 (0.5-0.9)	0.70 (0.5-0.9)	0.75 (0.5-1.0)	0.71 (0.5-0.9)	0.87 (0.6-1.1)	0.70 (0.4-0.9)
Active work/high strain	0.71 (0.5-0.9)	0.67 (0.5-0.9)	1.04 (0.7-1.3)	0.96 (0.7-1.3)	0.84 (0.6-1.1)	0.76 (0.5-1.0)
Social support at work						
Low	1	1	1	1	1	1
High	1.45 (0.9-2.2)	1.40 (0.9-2.1)	1.14 (0.7-1.7)	1.00 (0.6-1.5)	1.46 (0.8-2.4)	1.64 (0.9-2.7)

RR and respective 95%CI for the Poisson regression models; adjusted model: age and education level. All statistically significant associations are highlighted in bold (p < 0.05).

95%CI = 95% confidence interval; PA = physical activity; RR= relative risk.

## DISCUSSION

This study investigated the association between occupational stress and frequency of physical activity, as well as the association between social support at work and frequency of physical activity in adult workers of a university. The findings report an association between occupational stress and frequency of physical activity; however, the effect was different for men and women. The findings also highlighted an association between social support at work and frequency of physical activity among women. The literature shows that, even at similar positions, women are more prone to suffering with job strain when compared to men, as they are subjected to high demands of complex and stressful tasks both at work and in their personal lives, usually without financial compensation and institutional support. As women are more present in the current job market, this generates a labor burden. This way, they can perceive a higher influence of job demands on family matters and mental health,^19^ which can reverberate in taking up a physical activity. Social support at work is an important psychosocial resource that corresponds to the help obtained from colleagues and supervisors for performing work tasks, thus being considered an important aspect for minimizing possible health harms originated in the workplace.^20^ In addition, studies show that individuals who receive social support at work have a higher probability of being physically active.^21^

In this study, women classified as passive workers tended to have a lower risk of increasing the frequency of physical activity (walking) when compared to those with low strain. The characteristics of work can directly influence people’s lifestyles.^22^ Therefore, our hypothesis is that people performing passive jobs tend to adopt a more passive lifestyle, with low levels of leisure-time activities. In this sense, a cross-sectional study performed in California with the aim of investigating associations between occupational factors, obesity, and leisure-time physical activity in female nurses identified that high job demands were associated with increased regular aerobic physical activity. On the other hand, passive work was significantly associated with lower involvement in aerobic physical activities.^23^

In agreement with these findings, a cohort study performed with 170,162 workers showed that those who held passive or high-demanding jobs had a lower tendency of being physically active during their leisure time in relation to individuals at low-strain jobs.^24^ Evidence shows that a low job strain is considered the ideal occupational stress dimension for workers’ health, due to the low psychological demands and high decision latitude.^17^ Chou et al.,^25^ for example, when investigating the relationship between job strain and cardiovascular health in 1,329 doctors, observed that not only high strain was associated with a higher prevalence of cardiovascular problems, but workers in this category had 90% more chances of presenting low levels of physical activity in comparison to those who had low-strain jobs.

Among men, those classified as passive workers tended to have a lower risk of increasing moderate-intensity physical activity. This is in agreement with the literature: Gimeno et al.^22^ conducted a cohort study comprehending 6,085 participants (62.2% men), aiming to investigate the association between passive jobs and low levels of leisure-time physical activity. The findings showed that men who worked at passive jobs for approximately 5 years had 15% more chances of adhering to low levels of leisure-time physical activity than men in non-passive jobs, since even if job demands are low, the lack of stimuli and challenges at work end up influencing individuals into adhering to a passive lifestyle, especially regarding physical activity.

Curiously, in this study, active women with high-strain jobs tended to have a higher risk of increasing the frequency of vigorous-intensity physical activity when compared to those with low job strain. An important aspect is that, if work-related stress is relatively high, it can negatively affect the work, quality of life, and social functioning of an individual.^26^ However, for these women, physical activity may be an outlet, that is, vigorous-intensity behaviors serve as a measure for effectively dealing with the acquired stress.

The literature indicates that physical activity has a positive impact on occupational stress. Chen et al.,^27^ for example, when investigating the relationship between physical activity and occupational stress in Chinese workers, identified that a high level of leisure-time physical activity can be a protective factor against occupational stress. Another issue to be considered refers to the work characteristics of these women, as manual labor is attributed to a significant fraction of these participants; physical activity can thus be linked to physically demanding jobs. Conversely, active men with high-strain jobs tended to have a lower risk of increasing the frequency of walks and vigorous-intensity physical activity.

This is in consonance with previous findings, which show that individuals with higher levels of occupational stress tend to present lower adherence to physical activity.^28^ High job demands consume individuals’ time and energy, which can consequently be linked to less physical activity (both high-intensity and less demanding activities). Therefore, studies on the weekly frequency of physical activity become important for the working population due to the fact that individuals who exercise more frequently could have higher flexibility in the workplace.

As to social support at work, an association with the frequency of physical activity was found only among women. Those who were classified as having high social support at work presented a lower risk of increasing the frequency of moderate- and vigorous-intensity physical activity compared to those who had low social support at work. A possible explanation for this finding consists in the idea that, for these women, the psychosocial resource has a negative effect on the physical activity behavior; this resource can also not be enough for moderate- and vigorous-intensity physical activity, as high occupational demands tend to be an obstacle that overpowers adherence to this behavior. On the other hand, a Brazilian study including 11,779 public employees investigated the association between occupational stress and leisure-time physical activity and observed that social support at work reduced the probability of physical inactivity among women, highlighting the importance of support networks in occupational environments.^21^

This study presented some limitations that should be underlined. The study design was considered a limitation, since cross-sectional studies do not allow the observation of a cause-and-effect relationship as the investigated population is not followed up over time. Considering physical activity, although the validity and reproducibility of the IPAQ have been tested in 12 countries including Brazil,^29^ the scale has a subjective character, allowing individuals to overestimate their physical activity levels. Nevertheless, this fact was not considered a major problem for our study since we used the frequency of physical activity instead of its duration, and frequency is the domain least influenced by this limitation.

## CONCLUSIONS

Considering the benefits generated by physical activity to the physical and mental health of individuals, this study aimed to investigate the association between occupational stress and frequency of physical activity, as well as the association between social support at work and frequency of physical activity in adult workers. Based on our findings, it is possible to state that occupational stress is associated with the frequency of physical activity both in men and women. Moreover, social support at work was shown to be associated with the frequency of physical activity only among women. In this sense, new studies are required for analyzing these constructs more deeply within the studied population, especially with the aim of investigating the frequency of physical activity and not only the time spent doing these activities.
